# Study on the preparation of geranyl acetone and β-cyclodextrin inclusion complex and its application in cigarette flavoring

**DOI:** 10.1038/s41598-020-69323-1

**Published:** 2020-07-23

**Authors:** Fu Du, Tingting Pan, Xiaoming Ji, Jingyan Hu, Tianbao Ren

**Affiliations:** 1 College of Tobacco Science, Henan Agricultural University/Henan Province Flavors & Perfumes Engineering Research Center, Zhengzhou, 450002 China; 2 Hubei China Tobacco Industry Limited Company, Wuhan, 430030 China

**Keywords:** Chemistry, Analytical chemistry, Green chemistry, Medicinal chemistry, Organic chemistry, Chemical synthesis

## Abstract

β-Cyclodextrin (β-CD) inclusion complex containing geranyl acetone as a guest was prepared by saturated water solution method. Furthermore, the structure and properties of the inclusion complex were studied. The formation of the inclusion complex was demonstrated by. Fourier transform infrared spectroscopy (FTIR), X-ray diffraction (X-RD), thermogravimetric analysis (TG) and differential scanning calorimetry (DSC). The thermodynamic analysis of the inclusion complex showed that the inclusion reaction is an endothermic spontaneous reaction. The average of △H, △S and △G is 11.66 kJ mol^−1^, 0.082 kJ mol^−1^ and − 14.49 kJ mol^−1^, respectively. Moreover, the kinetic analysis of thermal decomposition of the inclusion compound showed that the thermal decomposition reaction is a first-order reaction (the inclusion ratio is 1:1), the average activation energy of the reaction is 180.90 kJ mol^−1^, and the binding force in the inclusion compound is mainly Van der Waals force. The flavor test of cigarettes showed that the inclusion compound improved the stability of geranyl acetone and the sensory quality of cigarettes. This study improves the solubility and thermal stability of geranyl acetone, and provides theoretical support and technical guidance for expanding the application of geranyl acetone.

## Introduction

Geranyl acetone (GA), scientific name 3,7-dimethyl-2,6-octadienyl acetone, is a kind of natural perfume with Magnolia fragrance, which has potential application prospects^1^. It is one of the main components of *Conyza bonariensis* L. (up to 25.3%)^2^, large yellow restharrow (20.3%)^3^ and field horsetail (13.7%) essential oils^4^. Because of its fresh and light floral fragrance with slightly sweet aroma of rose, it is widely used in daily chemical flavor enhancer^5^, food perfume fixative, and deployment of edible essence. Furthermore, GA has strong biological activity^6^ and antioxidant activity^7^, and it is used as a pharmaceutical intermediate and synthetic vitamin in medicine. Kawai^8^ found that it has a recovery effect on the heat shock response of gastric mucosa in malnutrition mice. GA has the ability to inhibit the growth of melanoma B-16 and leukae-mia HL-60 cell lines^9^. GA is a white or yellowish oily liquid at room temperature and difficult to dissolve in water and volatile, so that limits its application in the food industry and medicine. Therefore, improving the water solubility and stability of GA is the key to expand its application. Wang et al.^1^ studied the adsorption and slow-release effects of different adsorption materials on GA, and improved the stability of GA.

Cyclodextrin (CD) is a kind of conical cavity polymer composed of several d-glucopyranosyl units. It is predominantly divided into three products: α-CD, β-CD and γ-CD^10–12^, which contains six, seven and eight glucose units respectively. At present, the most widely used is β-CD, because it has a low production cost and moderate molecular void space, which is suitable for various fields^13,14^. The annual worldwide production of CDs exceeds 10,000 tons, of which ∼ 30% is used in pharmaceuticals, ∼ 20% for food industry, and the rest for various consumer products^15^. It has been pointed out that cyclodextrin can form noncovalent host–guest inclusion complex with a variety of molecules, including food additives^16–18^. In the inclusion system, the guest molecules penetrate the cavity of cyclodextrin, and they are mainly combined by Van der Waals force to form a relatively stable structure^19–21^. It has been emphasized that cyclodextrin inclusion complexes can increase the solubility of insoluble substances and the antioxidant activity of some drug molecules^22–27^, and improve the chemical stability and bioavailability of guest molecules^28–30^. Some studies also shown that cyclodextrin inclusion complex has a good sustained release effect^31^.

In this study, the inclusion complex of β-CD–GA was prepared by β-CD and geranyl acetone (GA). As far as we know, the β-CD–GA inclusion complex was synthesized and studied for the first time. The inclusion complex was distinguished by FTIR, X-RD and DSC. What’s more, the reaction thermodynamics and thermal decomposition kinetics of the inclusion complex were studied as well. The effect of inclusion complex on the stability of geranyl acetone was studied by cigarette flavor application.

## Materials and methods

### Reagents

GA (AR, ≥ 97%) was provided by Henan Xinzheng Jinye Flavor Co., Ltd (China), without further purification. β-CD (≥ 99.5%) was purchased from Sinopharm Chemical Reagents Co., Ltd (China). Absolute ethanol was purchased from Tianjin Kermel Chemical Reagents Co., Ltd (China).

### Equipment and instruments

UV-1800 ultraviolet visible spectrophotometer (Japan), Nicolet iS50 Fourier transform infrared spectrometer (America), D8 advance polycrystalline X-ray diffraction (Germany), NETZSCH STA 449 F3 simultaneous thermal analyzer (Germany).

### Preparation of β-CD–GA

Using saturated aqueous solution method prepared the β-CD–GA^24^. The GA was added into saturated β-CD solution in a molar ratio of 1:1 (β-CD: GA), and stirred them at 60 °C for 6 h. After the reaction, the obtained solution was slowly cooled to 4 °C and stood for 48 h. Then, the obtained solution was filtered to obtain white solid and washed repeatedly with deionized water. After freeze-drying, the white powder was β-CD–GA inclusion complex, which was stored in a sealed glass dryer for standby.

### FTIR

Four samples of β-CD, GA, MGA (the physical mixture of β-CD and GA (molar ratio 1:1) and β-CD–GA with KBr powder were respectively pestled to make a 1 mm thick sheet. Then the four kinds of tablets were analyzed by FTIR (Nicolet iS50). The scanning range was 4,000–400 cm^−1^ and the resolution was 4 cm^−1^.

### X-RD

Set the Cu-kα target λ = 1.54056 A, the working voltage is 40 kV, the current is 35 mA. Then take the appropriate amount of β-CD, MGA and β-CD–GA, scan in the range of 5°–50° 2θ, and the scanning speed is 0.02° min^−1^.

### DSC

Four samples of β-CD, GA, MGA and β-CD–GA were placed in the differential scanning calorimeter. The flow rate of carrier gas (high purity AR) was set at 20 mL min^−1^, the heating rate was 10 °C min^−1^, the heating range was 50 ~ 900 °C, and DSC was carried out.

### TG

The inclusion compound β-CD–GA was placed in the differential scanning calorimeter, and the carrier gas (high purity AR) flow rate was set at 20 mL min^−1^, and the temperature range was 50 ~ 900 °C. The inclusion compound was determined with the heating rates of 5 K min^−1^, 10 K min^−1^ and 20 K min^−1^, respectively.

### Drawing of the standard working curve of GA

Using UV-1800 ultraviolet visible spectrophotometer to scan the ethanol (anhydrous) solution of GA with the maximum UV absorption, it is found that there is a strong absorption peak at 279 nm. The results of repeated scanning showed that when the concentration of GA was 9.0 × 10^–3^ mol L^−1^, the absorbance was between 0.3 and 0.8. The concentrations of 6 × 10^–3^ mol L^−1^, 7.0 × 10^–3^ mol L^−1^, 8.0 × 10^–3^ mol L^−1^, 9.0 × 10^–3^ mol L^−1^, 1.0 × 10^–2^ mol L^−1^ and 1.1 × 10^–2^ mol L^−1^ GA solution were prepared respectively. The absorbance at 279 nm was measured. The standard curve was Y = 54.1277X + 0.00286 (R^2^ = 0.99782).

### Study on the application of cigarette flavoring with inclusion complex

Using the Yellow Crane Tower brand cigarette as the material, according to 1% feed ratio (100 g cut tobacco added 1 g inclusion complex), 0.500 g β-CD–GA, was weighed by analytical balance and dissolved in 10 mL 50% ethanol, then sprayed evenly on 50 g cut tobacco with a sprayer and balanced for 48 h under the condition of constant temperature (22 ± 1) °C and constant humidity (65% relative humidity). Then cut tobacco was taken out and made into cigarettes and stored in the refrigerator. The samples were sampled every 30 days for a total of 4 times, and the smoking was evaluated by experts from Hubei China Tobacco Industry Co., Ltd., in order to evaluate the stability of the inclusion complex in cigarettes. Cigarettes with the same quality of MGA were used as control.

## Results

### FTIR study of inclusion complex

**Figure 1:**
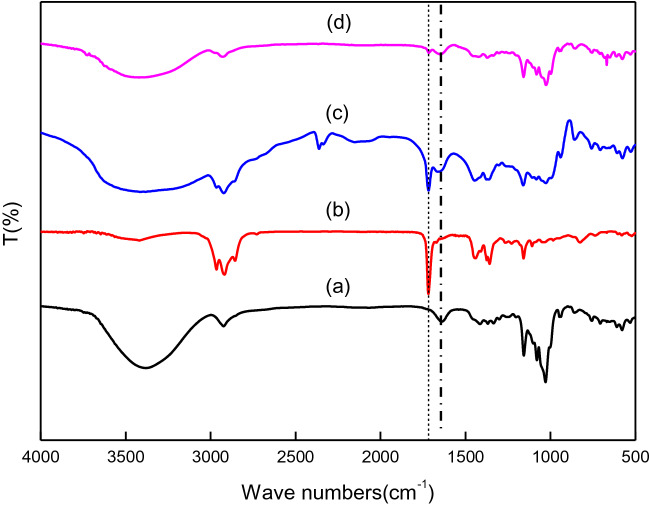
FTIR for: (a) β-CD; (b) GA; (c) MGA; (d) β-CD–GA.

The infrared spectra of β-CD, GA, MGA and β-CD–GA are shown in Fig. 1. The IR spectrum of β-CD (Fig. 3a) demonstrated crucial transmittance bands at ca. 3,300 cm^−1^ (O–H stretching), ca. 2,900 cm^−1^ (C–H stretching), ca. 1,600 cm^−1^ (H–O–H stretching), ca. 1,100 cm^−1^ (C–O stretching), and ca. 1,000 cm^−1^ (C–O–C stretching)^32,33^. In the curve (b), there is a strong v(C=O) stretching vibration at ca. 1,700 cm^−1^ frequency, which is the characteristic peak of GA. There are both the characteristic peaks of β-CD and GA in the curve (c), which is the simple addition of the two, indicating that MGA is only a simple mixture and does not form an inclusion complex. Compared with (c), the number of (d) peaks of the curve is obviously reduced, and the peak intensity is weakened. At ca. 1,700 cm^−1^ frequency, the characteristic peak of GA basically disappears, it might be attributed to that GA molecules were encapsulated into the cavity of cyclodextrin by hydrophobic force, hydrogen bonds and other secondary bonds, and restrained by the cavity^34^. The shape of the curve (d) is basically consistent with the shape of the curve (a) peak. It is suggested that GA has formed an inclusion complex with β-CD.

### X-RD study of inclusion complex

**Figure 2:**
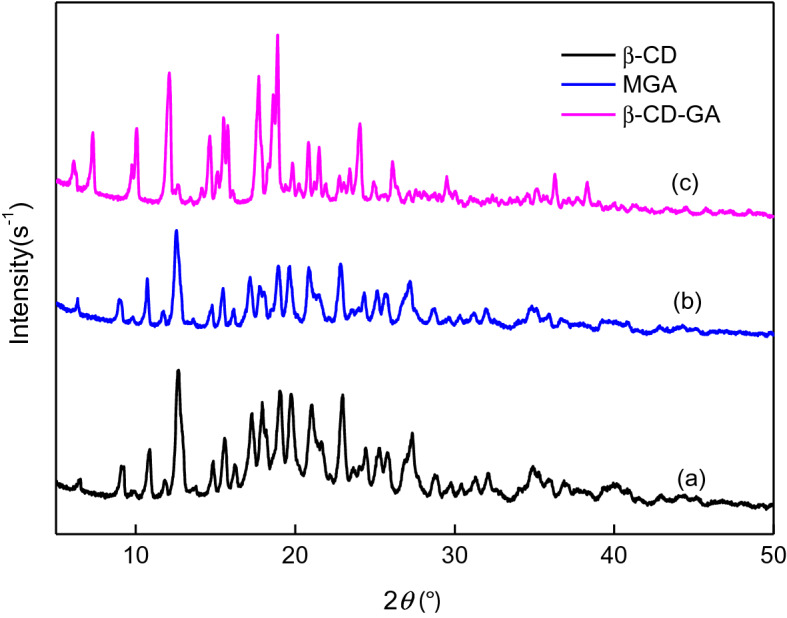
X-RD for: (a) β-CD; (b) β-CD–GA; (c) MGA.

As shown in Fig. 2a, the main diffraction peaks of β-CD were 6.2°, 9.0°, 10.7°, 12.5° (2θ)^34^, and the diffraction peak of the curve (c) shows the superposition of GA and β-CD. The curve (b) ceases to be a simple superposition of the two diffraction peaks, which is consistent with the curve (a) diffraction peak, but the peak intensity is weakened. Compared with the curve (c), the number of peaks decreased, the peak intensity weakened, and the characteristic peaks disappeared at 7.33° 2θ, 10.09° 2θ and 26.08° 2θ, the diffraction peaks of GA basically disappeared in the inclusion complex, while some weak new diffraction peaks were obtained, which might be due to the formation of inclusion complex of β-CD and GA^34^.

### DSC study of inclusion complex

**Figure 3:**
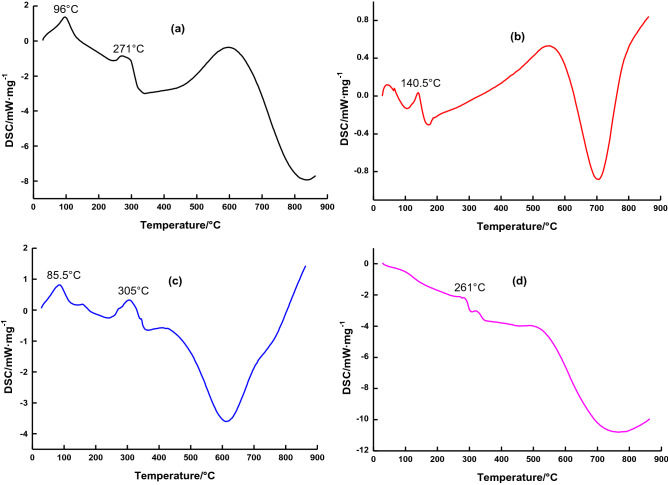
DSC for: (**a**) β-CD; (**b**) Geranyl acetone; (**c**) MGA; (**d**) β-CD–GA.

Due to the occurrence of interactions between the guest and the CD, the DSC curves shall present a not coincident profile with the sum of the effects observed in the thermograms of the constituents^35^, as shown in Fig. 3, β-CD has a characteristic endothermic peak at 96 °C and 271 °C correspond to the events of water loss and cyclodextrin decomposition, respectively^36^. GA has a characteristic endothermic peak at 140.5 °C, and MGA has both a characteristic peak of β-CD and a characteristic peak of GA, which is the addition of the two curves. It is merely that the intensity of the peak is weakened, indicating that MGA is only a simple physical mixture of the two. The DSC of physical mixtures at 85.5 °C and 305 °C is attributed to the dehydration and thermal degradation of β-CD, respectively. These changes have been confirmed^37,38^. However, β-CD–GA has a new endothermic peak at 261 °C, and the endothermic peaks of β-CD and GA disappear, the disappearance of the dehydration peak in the thermogram of the inclusion complex can be explained by the fact that the host molecule (GA) occupies the place of the water in the β-CD cavity, which proves the formation of inclusion complex β-CD–GA.

### Study on reaction thermodynamics

There is a Hildebrand Benesi relation for 1:1 inclusion system^39^:

1 $$\left[ {\text{G}} \right]_{{\text{T}}} /{\text{A }} = { 1}/{\text{K}}_{{{\text{CD}} \cdot {\text{G}}}} \cdot \, \left[ {{\text{CD}}} \right]_{{\text{T}}} \cdot \, \varepsilon \, + { 1}/\varepsilon$$

[G]_T_ and [CD]_T_ denote the total concentrations of GA and β-CD, respectively. ε is the molar absorptivity and K_CD·G_ is the stability constant of the inclusion complex.

**Figure 4:**
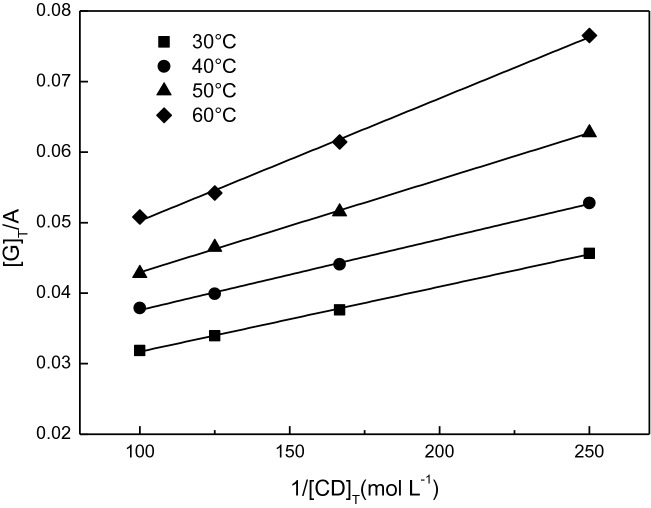
Relation between [G]_T_/A and 1/[CD]_T_.

Several parts of 9 × 10^–3^ mol L^−1^ GA solution (20% ethanol solution as solvent) were prepared, and 4.0 × 10^–3^ mol L^−1^, 6.0 × 10^–3^ mol L^−1^, 8.0 × 10^–3^ mol L^−1^ and 1.0 × 10^–2^ mol L^−1^ β-CD aqueous solution were added, respectively. Stirring at different temperatures (30 °C, 40 °C, 50 °C and 60 °C) for 6 h, the absorbance of the system was determined at 279 nm after inclusion equilibrium. The regression curves (Fig. 4) at different temperatures were obtained by using 1/[CD]_T_ as abscissa and [G]_T_/A as longitudinal coordinate. It can be seen from the diagram that there is a good linear relationship between [G]_T_/A and 1/[CD]_T_, indicating that the optimal molar ratio of β-CD to GA in the solution is 1:1. Sambasevam^23^ and Wang^40^ also found the same results. The stability constants (Table 1) of the inclusion complex at 30 °C (303 K), 40 °C (303 K), 50 °C (303 K) and 60 °C (303 K) can be obtained from the slope and intercept of the straight line. With the increase of temperature, the stability constant of the inclusion complex increases, indicating that the inclusion process is an endothermic reaction.

**Table 1:** Curve equations of [G]_T_/A and 1/[CD]_T_ at different temperatures.

Temperature (K)	Fitting equation	R^2^	K
303	Y = 0.000174X + 0.03288	0.99734	1.89 × 10^2^
313	Y = 0.000132X + 0.02979	0.99912	2.26 × 10^2^
323	Y = 0.000100X + 0.02591	0.99792	2.58 × 10^2^
333	Y = 0.000090X + 0.02647	0.99886	2.87 × 10^2^

**Figure 5:**
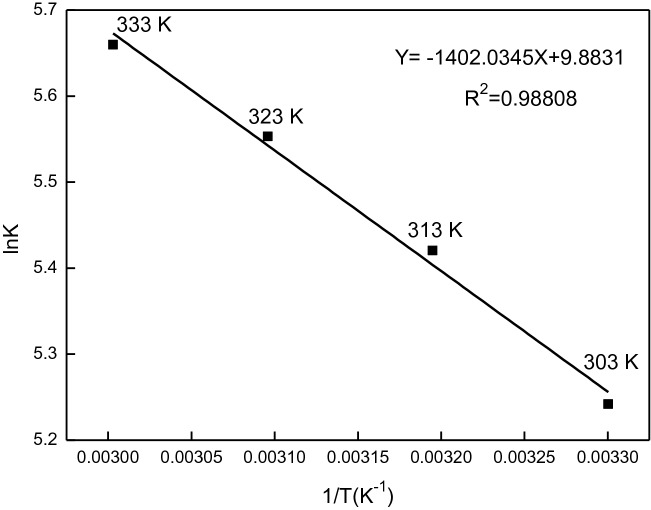
The relation of lnK and T^−1^ in different temperature.

According to the relational formula lnK = − ∆H/RT + ∆S/R, the lnK of β-CD and GA inclusion system is used to map T^−1^ (Fig. 5). According to the slope and intercept of the straight line and the relation ∆G = − RTlnK, the ∆G, ∆H and ∆S of the inclusion system at different temperatures can be calculated (see Table 2). The average ∆H, ∆S and ∆G of the reaction are 11.66 kJ mol^−1^, 0.082 kJ mol^−1^ and −  14.49 kJ mol^−1^, respectively. From ∆H > 0 and ∆S < 0, it can be seen that the inclusion reaction is a spontaneous endothermic reaction.

**Table 2:** Stability constants and ΔG of β-CD–GA at different temperatures.

Temperature (K)	K	∆*H* (kJ mol^−1^)	∆*S* (kJ mol^−1^)	∆*G* (kJ mol^−1^)
303	1.89 × 10^2^	11.69	0.082	− 13.25
313	2.26 × 10^2^	11.61	0.082	− 14.11
323	2.58 × 10^2^	11.63	0.082	− 14.91
333	2.87 × 10^2^	11.69	0.082	− 15.67

### Study on thermal decomposition kinetics

**Figure 6:**
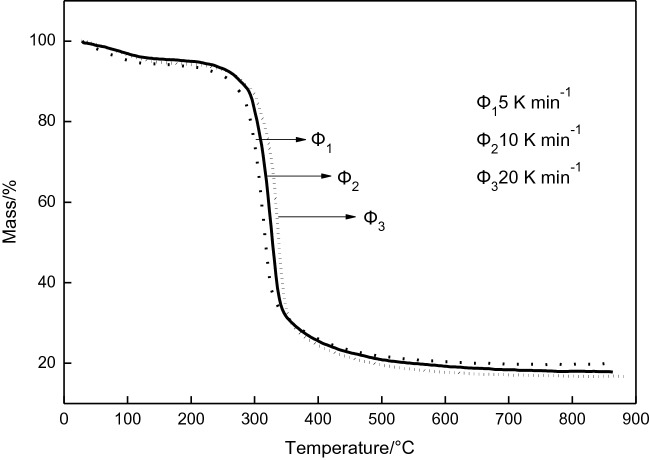
TG curves of β-CD–GA in different heating rate.

The thermal decomposition TG curves of β-CD–GA at different heating rates are shown in Fig. 6. It can be seen from Fig. 6 that different heating rates have an effect on the decomposition (weight loss) rate of the inclusion complex, and a higher heating rate can promote the thermal decomposition reaction. There is a relationship for simple thermal decomposition^41^:

2 $${\text{lnln}}\left( {{1}/\left( {{1} - \alpha } \right)} \right) \, = \, - E/R{\text{T }} + {\text{ b}}$$

The kinetics of any solid-state decomposition reaction can satisfy the relation of Flynn and Wall^42^ and Ozawa^43^:

3 $$\log \Phi = \, \log ( - AE/Rf(\alpha )) - 2.315 - 0.4567\left( {E/R{\text{T}}} \right)$$

When n = 1, the relation (3) can be converted to:

4 $$\log \Phi = \, \log ( - AE/R\ln (1 - \alpha )) - 2.315 - 0.4567\left( {E/R{\text{T}}} \right)$$

In (2), (3) and (4), α is the mass loss rate, b is the constant, *A* is the pre-exponential factor, T is the temperature, *E* is the activation energy, *R* is the gas constant, and *Φ* is the heating rate. When the thermal decomposition reaction of the inclusion complex is a first-order reaction, lnln (1/(1 − α)) is a straight line for 1. In (4), *f*(*α*) = ∫d*α*/(1 − *α*)^n^, (n is the reaction order).

**Figure 7:**
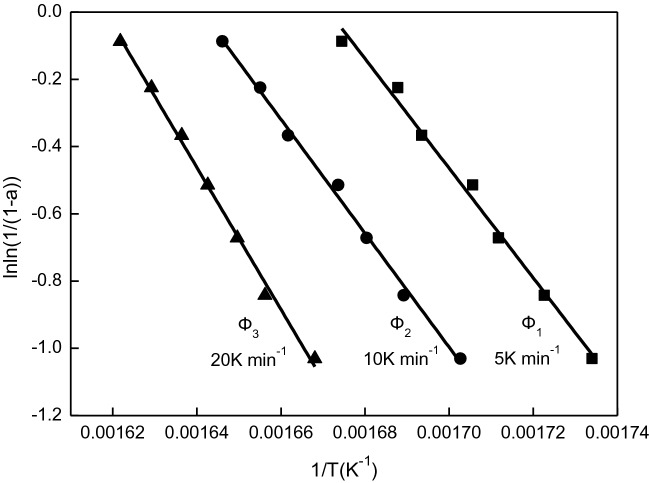
The relation of [lnln (1/(1 − α))] and (1/T) in different heating rate.

**Figure 8:**
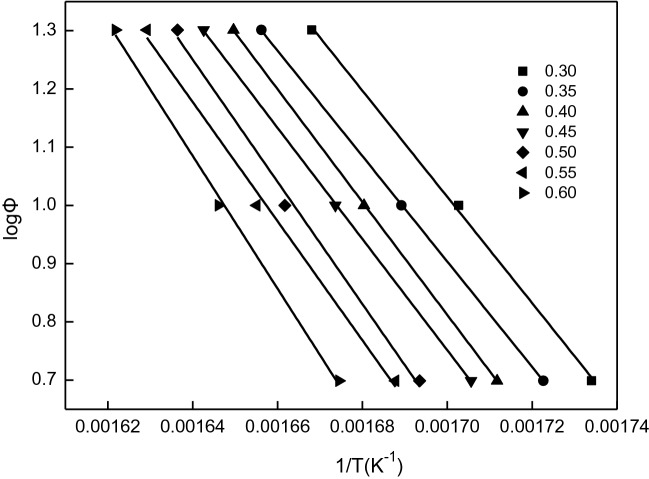
The relation of log*Φ* and (1/T) in different weightlessness rate.

Table 3 lists the corresponding temperature values of the inclusion complex at the same weight loss rate (α) at different heating rates (5 K min^−1^, 10 K min^−1^ and 20 K min^−1^). The median of Table 3 was brought into the relation formula (2), and the lnln (1/(1 − α)) of the inclusion complex was plotted at three heating rates, and the Fig. 7 was obtained. It can be seen from Fig. 7 that, lnln (1/(1 − α)) has a good linear relationship with 1 at different heating rates, indicating that the thermal decomposition reaction of inclusion complex β-CD–GA is a first-order reaction. At the same weight loss rate, α is constant, and the lines in the Fig. 8, diagram obtained by linear quasi-combination of 1 and log*Φ* have a satisfactory linear relationship. According to Fig. 7, the kinetic parameters of thermal decomposition of the inclusion complex can be obtained in Table 4, and the activation energy *E* and pre-exponential factor *A* are calculated according to the slope and intercept of the straight line.

**Table 3:** Temperatures corresponding to the same mass loss in different heating rate.

α	T(K)		
	5 (K min^−1^)	10 (K min^−1^)	20 (K min^−1^)
0.30	576.70	587.30	599.50
0.35	580.50	592.00	603.80
0.40	584.20	595.10	606.20
0.45	586.30	597.50	608.80
0.50	590.50	601.80	611.10
0.55	592.20	604.20	613.80
0.60	597.20	607.50	616.60

**Table 4:** The kinetic parameters of thermal decomposition of β-CD–GA.

Sample	α	Slope	Intercept	*E* (kJ mol^−1^)	*A*
β-CD–GA	0.30	− 9,121.57	16.52	166.05	1.22 × 10^14^
	0.35	− 9,050.75	16.30	164.87	8.95 × 10^13^
	0.40	− 9,691.29	17.29	176.43	9.63 × 10^14^
	0.45	− 9,550.40	16.99	173.86	5.72 × 10^14^
	0.50	− 10,500.88	18.47	191.16	1.85 × 10^16^
	0.55	− 10,233.60	17.96	186.30	6.72 × 10^15^
	0.60	− 11,404.80	19.79	207.62	4.67 × 10^16^
Average activation energy *E* (kJ mol^−1^)				180.90	

In the non-isothermal thermal decomposition reaction, the values of *E* and *A* decrease with *Φ*, which can be characterized by the kinetic effect of solid-state thermal decomposition. The law of dynamic compensation effect is log*A* = k*E* + b (k, b is the dynamic compensation constant). The mathematical expression of dynamic compensation is log*A* = 0.08644*E* − 0.2670 by substituting the data in Table 4. The average activation energy of thermal decomposition of β-CD–GA is 180.90 kJ mol^−1^ and the pre-exponential factor is 1.05 × 10^16^. The lower apparent activation energy indicates that there is no strong chemical bond between β-CD and GA, which is mainly due to the combination of Van der Waals force.

### Study on the flavor stability of cigarette with inclusion complex

**Figure 9:**
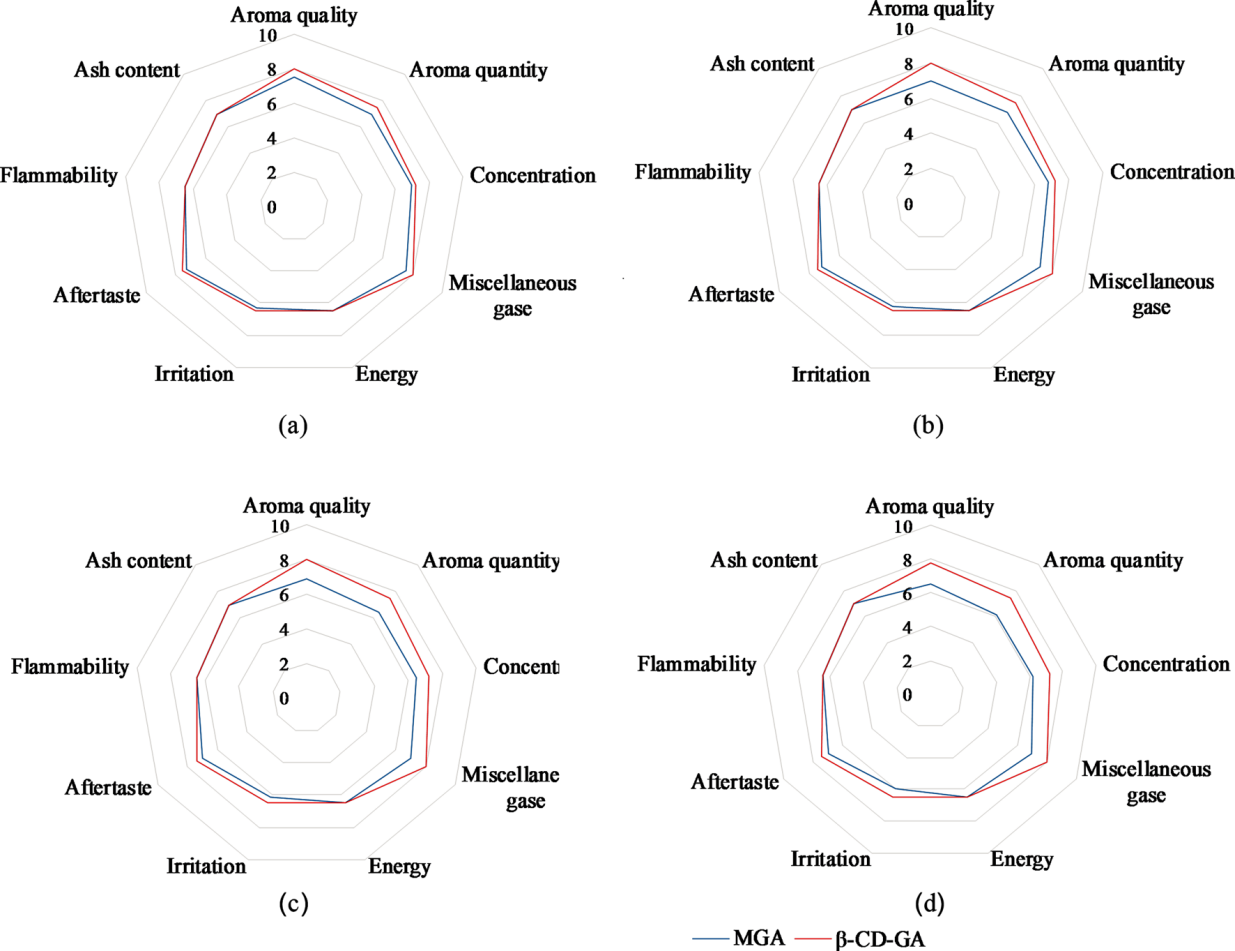
The sensory quality score of cigarettes added with MGA and β-CD–GA varied with storage time: (**a**) 0 day; (**b**) 30 days; (**c**) 60 days; (**d**) 90 days.

The scores of sensory quality indexes of cigarettes with MGA and β-CD–GA changing with storage time under constant temperature and humidity are shown in Fig. 9. It can be seen that there is no significant difference in sensory quality between the two kinds of cigarettes after being placed for 0 day under the condition of constant temperature and humidity, which indicates that the preparation of geranyl acetone inclusion complex does not affect the sensory quality of cigarettes. However, with the increase in storage time, there was a great difference in the sensory quality of the two kinds of cigarettes, and the difference reached the maximum at 90 days. The addition of inclusion complex mainly improved the aroma quality and quantity of cigarettes, reduced miscellaneous gases and irritation, and improved the coordination of cigarette smoking evaluation. After 90 days of storage, the score of sensory quality of cigarettes with β-CD–GA was basically unchanged. However, with the passage of time, the sensory quality scores of cigarettes with MGA gradually declined, which was not conducive to cigarette smoking. The above results show that β-CD–GA has strong stability in cigarette flavoring and improves the sensory quality of cigarettes. The results also proved that the inclusion complex could reduce the volatility of geranyl acetone and improve its stability.

## Discussion

In this study, β-CD was utilized as an embedding material to prepare β-CD–GA. A series of analytical results of FTIR, X-RD and DSC spectra proved the formation of the inclusion complex. By studying the thermodynamics of the inclusion complex reaction, it is found that the formation of the inclusion complex is a spontaneous endothermic reaction, and the stability constants at different temperatures are obtained. The study of the thermal decomposition kinetics of the inclusion complex shows that the thermal decomposition reaction of the inclusion complex β-CD–GA is a first-order reaction, indicating that β-CD and GA combine to form the inclusion complex at 1:1, and the average activation energy of the thermal decomposition reaction is 180.90 kJ mol^−1^. The main force between β-CD and GA is the Van der Waals force. The inclusion complex can improve the stability of geranyl acetone in cigarette flavor and improve the sensory quality of cigarette.

In conclusion, the formation of the inclusion complex improves the solubility and stability of geranyl acetone. This study can provide guidance and basis for the application of geranyl acetone in food processing, medical medicine, cigarette formula flavor and other industries.
